# Online psychotherapy: trailblazing digital healthcare

**DOI:** 10.1192/bjb.2019.66

**Published:** 2020-04

**Authors:** Annabel McDonald, Jessica A. Eccles, Sanaz Fallahkhair, Hugo D. Critchley

**Affiliations:** 1 Brighton and Sussex Medical School, UK; 2 School of Computing Engineering and Maths, University of Brighton, UK

**Keywords:** Internet, online, psychotherapy, access

## Abstract

Advances in digital technology have a profound impact on conventional healthcare systems. We examine the trailblazing use of online interventions to enable autonomous psychological care which can greatly enhance individual- and population-level access to services. There is strong evidence supporting online cognitive–behavioural therapy and more engaging programmes are now appearing so as to reduce user ‘attrition’. The next generation of autonomous psychotherapy programmes will implement adaptive and personalised responses, moving beyond impersonalised advice on cognitive and behavioural techniques. This will be a more authentic form of psychotherapy that integrates therapy with the actual relationship experiences of the individual user.

As in many other countries, the UK is looking to expand digital technology to control burgeoning healthcare costs. These plans are articulated in recent government publications such as *The NHS Long Term Plan*.^1^ The top level of this ambitious scheme refers to a system that might offer seamless care across all aspects of the National Health Service (NHS) and associated services. A second level proposes enhancing access to clinicians though digital platforms such as video conferencing channels. A third level is the ambition for broad implementation of technological monitoring services for patients in their homes, enhancing care in the community through high-quality information. However, this long-term plan also illustrates that, particularly in general medicine, the implementation of autonomous computerised treatment remains a distant goal. In mental health, by way of contrast, psychotherapy is trailblazing this field. The recent Royal College of Psychiatry recommendations accompanying the NHS long-term plan^2^ recognise the value of extending therapist-led psychotherapies through technology, for instance by using avatars to explore client identity. However, even here there is limited recognition of progress toward autonomous psychotherapy treatments where digital treatment programmes are established NHS-approved referral options. Examples of such programmes include *FearFighter* (UK),^3^ *MoodGYM* (Australia; https://moodgym.com.au/) and *SPARX* (New Zealand; https://www.sparx.org.nz/). We refer to these programmes in our appraisal of current digital psychotherapy services, which we then compare with the next generation of autonomous therapies that will offer a truly individualised form of therapy, informed by the user's personal profile and experience.

## Advantages associated with an online medium for psychotherapy

Online therapy offers a number of potential advantages compared with other therapies. Perhaps the most obvious are broad accessibility and low treatment cost; however therapists also refer to the fact that patients often find it a less inhibiting medium, enabling greater disclosure and interaction.^4^ There is, in addition, a belief that young patients are generally more willing to engage with digital interfaces, as explicitly noted by the team working on a gaming application in New Zealand (*SPARX*).

### Accessibility

Specific groups may particularly benefit from the improved accessibility of psychotherapy when offered through an online medium. These include those in low- and middle-income countries, the financially disadvantaged individuals without access to a free health service, full-time employees, people who cannot physically visit therapists and those who are not deemed ‘ill enough’ to meet a threshold to be offered services. Nevertheless, accessibility would not be augmented for those who are unable to engage with a digital service due to a lack of equipment, physical constraints or poor online confidence. Such individuals are over-represented in psychologically vulnerable groups.

In low- to middle-income countries, the responses to psychotherapy for depression are high, and there does not appear to be a requirement for the therapy to be adapted to local situations.^5^ These effects appear larger than in the high-income countries; this may partly reflect less-efficacious ‘standard treatments’, resulting in limited improvement in comparison control groups. There is ample scope to access the online-therapy format as ownership of mobile phones has high priority in these communities: 67% of African people owned a mobile phone in 2018.^6^

In high-income countries, free provision of psychotherapy is by no means universal. Psychotherapy is a relatively expensive service, often provided on an individual basis in predefined sessions typically totalling 6–20 h. Financial costs limit access to those who can afford treatment but, even where free-to-user services exist, access tends to be rationed according to symptom severity and degree of functional impact. Thus, individuals who are functioning reasonably well, in particular those who are working, are rarely eligible for psychotherapy even if quality of life is low. An online service could reach many of those individuals who cannot afford high therapy costs, and those individuals judged to be below the arbitrary threshold of clinical severity for access to standard delivery of psychotherapy.

Physical constraints compromise engagement with multisession therapies and may have medical, social or psychological origins (e.g. physical disability, imprisonment or agoraphobia). Among mental health symptoms, Community Mental Health Teams in the UK recognise agoraphobia and social anxiety as a common barrier to accessing and delivering therapy. However, community patients can also struggle to access psychotherapy if they are perceived as having anger problems and are implicitly judged a risk as emotionally challenging issues are explored during therapy. Large geographical areas with sparse populations can limit access to specialised psychotherapy. In Australia and New Zealand, geographical access has motivated the priority development of online psychotherapy.

### Financial costs

Online psychotherapy encompasses multiple modalities and approaches, including online contact – either through a video service or texting – with a therapist. Clearly the cost of this type of treatment is broadly comparable to face-to-face therapy in well-populated areas where travel is not a major factor but it can support service access. There are also systems that offer a limited amount of personal contact with a guiding therapist while the patient works through online modules. This can take the form of ‘check-up’ phone calls through to full therapy sessions interspersed between the computerised modules. However, ‘unguided’ therapy, where the patient uses a programme autonomously without any direct or personalised therapist support, has the most potential to save significant costs. Autonomous digital psychotherapy can nevertheless incorporate automated text messages and even user forums, while retaining minimal therapist costs. This type of therapy may represent a viable ‘first-level’ care strategy, with patients progressing to therapy with personal contact if required thereafter.^3^ Many programmes have been shown to be effective; however sustaining patient engagement in the therapy, which can be as low as 20% by the end of a treatment programme,^7^ remains a problem. However, because such a large number of people could be treated very cheaply, the potential gain in care is still enormous, provided the online therapy does not discourage or prejudice the efficacy of any future personalised therapy.

## Current online therapies

The aim of early pioneering work on online therapy was to overcome access problems caused by population dispersal. Leading developments have thus originated in Australia (e.g. *MoodGYM* and *BluePages* [https://bluepages.anu.edu.au/]). Similarly, in New Zealand the development of *SPARX* was driven by poor mental health across widely dispersed young people. These therapies are offered on three financial bases: charged, access paid by health services and free to access. Australian services approve the use of *MoodGYM* (free) and *MyCompass*.^8^ New Zealand nationals can use *SPARX* for free. The UK's NHS partly funds the use of two computerised cognitive–behavioural therapy (CBT) programmes, *Beating the Blues* (http://www.beatingtheblues.co.uk/) and *FearFighter* (https://magellanascend.com/Content/View/2526), as part of their 'stepped model' of treatment for depression and anxiety, respectively.^3^

**Fig. 1:**
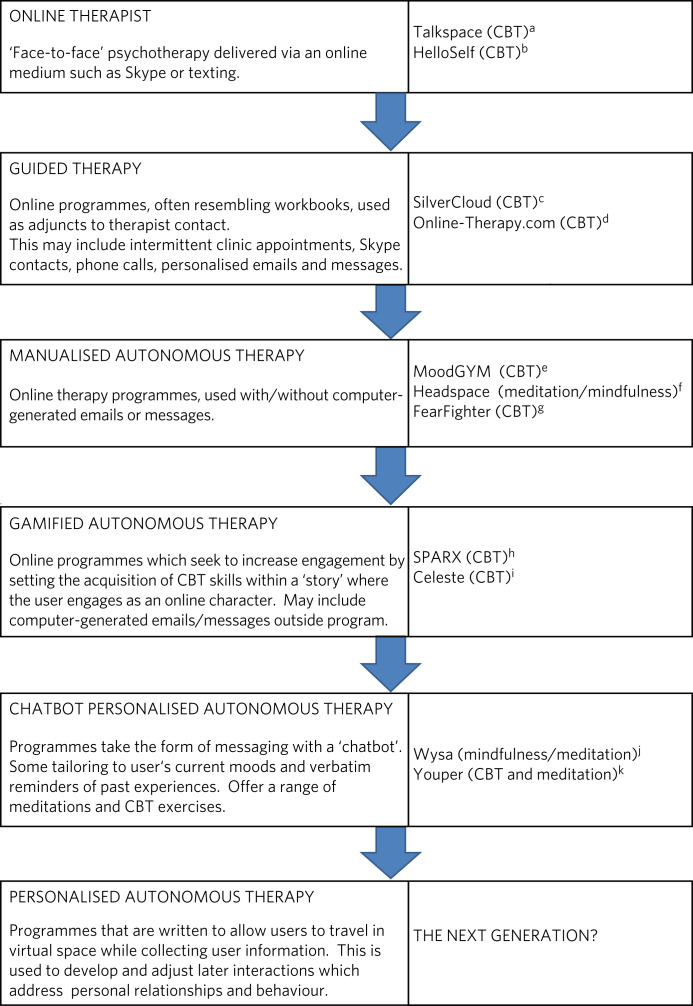
Evolution of online autonomous psychotherapy (may include access to a user forum). a. https://business.talkspace.com/?_ga=2.6168373.769027487.1553093877-2135218530.1553093877 b. https://www.helloself.com/ c. https://www.italk.org.uk/1-2-1-treatments-groups/computerised-therapy-c-cbt/ d. https://www.online-therapy.com/about_us.php e. https://moodgym.com.au/ f. https://www.headspace.com/ g. http://ccbtmain.cbtprogram.com/products/fearfighter/ h. https://www.sparx.org.nz/ i. http://www.celestegame.com/ j. https://www.wysa.io/meet-wysa k. https://www.youper.ai/about-us

The evolution of online psychotherapy is shown in Fig. 1. The earliest autonomous treatments, primarily based on CBT, are interactive programmes where users complete questionnaires and white-space areas with their own information so as to develop their insight and encourage internal reflection about habitual behaviour. This is combined with examples of characters with extreme forms of behaviour to help the user recognise their own thinking and behaviour patterns. *MoodGYM* is an illustrative example of this form of online therapy. Extensive evaluation suggests *MoodGYM* promotes significant improvements in users’ mental states.^9–13^ *MoodGYM* and related programmes generally consist of five to ten CBT modules that consider key aspects such as negative thinking and activation. The path through the programme is not modified according to earlier information provided by the user. This genre has now been extended to programmes that engage the user in mindfulness and meditation. *Headspace* (https://www.headspace.com/) is a well-known commercial example. Here the user participates in meditation exercises, including breathing and focusing, accompanied by calming graphics. Programmes within this general category, including those based on both CBT and mindfulness, may send automated texts to the user, often as reminders to engage with the programme. There is also a subgenre that combines online therapy with intermittent therapist contact through the use of phone calls, messages or emails.

CBT, cognitive–behavioural therapy.

These early forms of online psychotherapy treatment require a high level of motivation that may be challenging for people with mental health problems. Concerns about engagement have prompted the introduction of ‘gamified’ CBT resources such as *SPARX* and *Celeste* (http://www.celestegame.com/), where CBT techniques are embedded within an intrinsically entertaining experience. *SPARX* offers self-help for young New Zealanders by being set within such a video-gaming framework. The user selects an avatar to fulfil a number of tasks, which include challenges such as bursting floating bubbles called GNATS (gloomy negative automatic thoughts) with a staff, while receiving CBT-based wisdom from a guiding character who draws links to behaviour in the user's own life. *Celeste* is an even more traditional game which has drawn acclaim from video-game forums. *Celeste* centres on a nervous character who ‘learns’ to modify anxiety through CBT techniques that include breathing- and meditation-style tasks. These programmes may herald a new genre in which online virtual worlds combine gaming with the mutual support of internet forums.^14^ *Autcraft* (https://www.autcraft.com/) is another notable example of an early development of this format; it is packaged as a ‘limited edition’ of Minecraft which offers a kinder and more protected environment for autistic users to build confidence and practice social interaction. There is, however, a tension within such gamified applications in balancing the need to provide therapy while maintaining the unbroken flow of a game.

Not unrelated to interactive gamified therapy is the increasing presence of ‘chatbot’ forms of therapy. The programme is set within a machine-messaging environment and so is able to ‘chat’ to the user. These programmes, of which *Wysa* (https://www.wysa.io/meet-wysa) is a very good example, tend to deliver CBT, mindfulness and meditation. User are free to move between the encouraging and supportive online machine ‘conversations’ and subunits engaging them in direct psychotherapy. These programmes store information about the user, including their current state of mind, and use this information within the ongoing conversation. An example of this would be a comment such as ‘You said that you felt low yesterday, are you feeling a bit better today?’

The next stage in the evolution of autonomous online therapy is likely to involve a significant extension of the collection of user information and increased ‘tailoring’ of programme responses to the user's behaviour and relationship experiences (Fig. 1). This would move online care beyond CBT-style interventions and start to offer a relational form of therapy, through which the user can develop a deeper insight into his/her own relationship styles, their aetiology and potential means of escaping negative patterns of behaviour.

Existing internet forums can offer mutual support for those with psychological distress or more specific mental health problems. They are sometimes suggested or offered as adjuncts to the types of digital therapy programmes described above. The use of such forums may or may not be free and are generally moderated. Examples include *Mood Garden* (http://www.moodgarden.org/) and *Big White Wall* (https://www.bigwhitewall.com/v2/Home.aspx?ReturnUrl=%2f), the latter of which has trained staff online on a 24-h basis.

## Evidence base for online therapies

Research into online therapies reveals two important aspects: (1) improvement of psychological state, and (2) the degree of engagement demonstrated by users (Supplementary Table 1 available at https://doi.org/10.1192/bjb.2019.66). Perhaps unsurprisingly in view of the heterogeneity of studies, a wide range of results are shown for both of these measures across the different online programmes. The selection of participants is also a factor, which may depend upon unsolicited clicks on a website through to targeted selection of a specific patient group. Many studies include brief phone or text contact to encourage engagement.

Online CBT programmes have been the main source of evidence for efficacy of digital therapies. However, examination of bias-modification programmes for anxiety reveals weak effects of borderline significance (observed effect sizes, 0.07–0.42).^8^ In contrast, for a mindfulness programme aimed at patients with established bipolar disorder, a significant change is observed with an effect size of 0.52 on an intention-to-treat basis. Nevertheless, the attrition rate was 38%.^15^

There is a paucity of published evidence on those users who begin online CBT therapy independently through search engines or clinical recommendations. One informative exception is data on the use of the modular CBT programme *MoodGYM.* Only around 25% of arrivals proceeded from the initial introductory module through to a second module. Moreover, the maximum observed pre-post effect size was 0.4.^7,9^ Notably, around 50% of those enrolling on such programmes fulfil clinical criteria for depression.

Thus the majority of research into effectiveness examines the effects of CBT programmes on preselected clinical populations. Here, the typical pre-post effect size is 0.5–0.8^8,13,16,17^ with variation between individual studies. The effect size drops when a comparison group – typically a treatment-as-usual or waiting-list group – is included, negating statistical group differences in a subset of studies. On average, the effect size is typically reduced to around 0.4,^13,17,18^ which represents a low to moderate treatment effect. This suggests that part of the pre-post effect is a natural recovery cycle from psychological distress, a view also supported by mixed findings as to whether recovery is enhanced by an increased engagement with the digital programme. No association was found between reliable clinical improvement and either the (extended) duration of engagement with an online programme^18^ or the number of therapy modules offered,^7^ although other studies do report increasing therapeutic benefit in association with longer therapy engagement.^19^

Engagement is generally poor, with high rates of attrition among user of online therapies. A true meta-analysis of these data is impossible given the range of measures across studies (including full programme completion, minimum ‘adequate’ number of therapy sessions, average percentage of completed modules, percentage of participants progressing to second module or percentage completing half of the modules). However, data for full completion or adequate engagement show a wide range, e.g. 16–82% for completion. A ‘typical’ value appears to be around 50% for the completion of half of the modules.^12,13,17,18,20–25^

The degree of engagement with online therapy is likely to reflect the variety of programmes, the range of indications or whether users were contacted (e.g. brief emails or text) to encourage adherence. Some of the factors that affect therapy engagement have been studied; for example observed higher levels of therapy adherence are reported in people with lower baseline symptoms.^19^ Although other studies have not found associations between engagement and symptom severity or improvement.^18^ Among social factors, adherence is reported to be higher among users who are white and older.^23^ A meta-analysis also found engagement is better in females, individuals with higher educational attainment and in older users. Comorbid anxiety symptoms appear to introduce an additional challenge to adherence.^26^

## Forms of additional support for online therapy

A number of ‘add-ons’ have been introduced to stand-alone online therapy programmes, generally with the aim of reducing attrition. These include brief contact with therapists or allied health staff by phone or text. The use of weekly phone calls seems to be a particularly popular approach during treatment trials with the aim of increasing engagement. The number of therapy modules, out of ten, that were completed increases from when a user has no contact, to a weekly email to a weekly phone call (3.7, 5.5 and 7.3 modules, respectively).^24^ A related approach is the use of automated reminder emails. Interestingly, better results appear to be achieved when the automated email informs the user about new site content, rather than simply reminding them to return to the programme. Moreover, email reminders are more effective when sent after 2 weeks of absence than when the user had been absent for 4 or 6 weeks.^27^

An alternative add-on is membership of a social forum with other users, noted above to be a potentially valuable adjunct to a bipolar disorder mindfulness programme.^15^ Although the addition of a forum generally requires staffing in the form of moderation, peer support can improve adherence to psychoeducation modules.^28^ Forum membership is reported to provide an impetus to ‘keep going’ in a qualitative study of a CBT sleep improvement programme. Here, users offered each other support during difficult parts of the programme.^29^ Reported reasons for involvement with the forum were a desire to connect with peers, receive personalised advice, curiosity, being invited and wanting to use all sleep improvement tools. Reasons given for not joining the forum include design problems, negative self-comparisons, excessive time commitment, data privacy concerns and the uncertain quality of user-generated content. A user forum linked to an online CBT programme for individuals receiving prostate cancer treatment failed to show any improvement in CBT programme completion.^30^

## Online therapy: strengths, weaknesses and potential concerns

As previously discussed, there are concerns about the high attrition rate in the use of online therapy programmes and the small-to-moderate effect size when compared with other groups such as those on the waiting list or receiving treatment as usual. Expectation management should thus form an integral part of such programmes.^31^ One potentially compensating effect at the service level is the low cost of massive open online interventions. When the cost of minor improvement is minimal and the number of patients receiving treatment is so large, there is a large resulting gain in psychological health. Related to this is the advantage, highlighted above, afforded by an increased accessibility of treatments that no longer need to be time limited.

Online therapy is likely to be most appropriate as an early phase in a stepped treatment plan.^3,13^ A potentially worrying aspect of online therapy is whether it might have a negative impact on acceptance/receptivity to face-to-face therapy. Interestingly, enthusiasm for personal CBT may be increased among individuals receiving online treatment when compared with the provision of only psychoeducational information about depression,^32^ or there may be no observable effect on face-to-face treatment.^33^ Some users of course may become ‘well enough’ that they do not feel in need of the further improvement that could occur through subsequent face-to-face therapy.^34^ This is an odd reversal of the potential gain of online therapy improving the condition of those not considered ill enough to require face-to-face therapy.

It is also important to consider whether other harmful effects might result from engaging in online psychotherapy. Higher rates of clinical deterioration occur in patients receiving watchful waiting (7.2%) than those receiving online CBT (5.8%).^35^ Feedback from those who completed a mindfulness programme saw 15 users denying any issues whereas 1 had been uncomfortable during a 30-min exercise (body scan) as it reawakened a traumatic memory.^15^ This potentially supports the concern that re-traumatisation might occur with increased vulnerability due to the reduction of psychological defences. A less direct form of harm to a user might occur if online responses could be subject to subpoena.^34^

There are potential concerns that high-risk individuals may not be identified during online therapy. Questions have been raised as to whether it should be possible, or ethical, to trace individuals directly if worrying information is declared while using the programme. Coupled telephone help services are provided for users of some programmes such as *SPARX*. Other programmes rely on the presentation of emergency contact details in a more generic form, for example advising users to contact local mental health services or helplines. Similar issues have long been recognised with respect to self-help manuals, whether they are presented as literature or online. Online therapies have more opportunity to provide support through ‘help’ buttons or facilities to put the user in immediate contact with personal support by phone, email or messaging. An as-yet-unresolved issue relates to the international nature of the internet that makes it difficult to establish the jurisdiction under which the programme is being operated,^34^ and consequently how users might seek redress for grievances.

## The next generation

All of the psychotherapy programmes discussed above share a critical limitation. They do not adapt to the behaviour and relationship styles of the individual user. They set out to treat a ‘typical psychology patient’ and supply information that is known to be widely helpful in developing a healthier self-narrative. Thus *MoodGYM* encourages the user to understand the concept of ‘warpy thoughts’, which are related to automatic negative assumptions. *MoodGYM* illustrates this principal with tales of the experiences of programme characters with varying mental states. The user is then invited to reflect on circumstances in their own life when they react in this automatic negative manner. There is no feedback or onward development of these personal experiences by the programme. Similarly, *SPARX* encourages the user's avatar to destroy pictorial bubbles or GNATs, then further expands on how these types of thoughts are expressed in ‘real life’. But again, the information is not specific to the user's experiences in any manner. The chatbot programmes provide generic information for problems suggested during the chat conversation.

The limited dynamic personalisation makes it very difficult to move beyond meditation- and CBT-type programmes toward more relational forms of psychotherapy that are critically dependent on personal interactions both inside and outside the therapy room. Nevertheless, there is no inherent reason why such relationships cannot be explored online with personalised responses. The programming of such a functionality within a digital therapy is inherently more complex, requiring a wider range of outcomes, depending on the user's inputs. The implementation of such levels of complexity within online psychotherapy programmes is ongoing and is anticipated to lead to truly personalised therapies. This key development will presage the arrival of the next generation of autonomous online psychotherapy programmes.

## Future directions

We are at an exciting phase in the development of autonomous online psychotherapy services. Increasingly, programmes are aiming to move from being informative to entertaining. The advent of programmes such as *SPARX* and *Celeste* as well as the protected virtual reality of *Autcraft* show how developers now attempt to offer integral enjoyment to facilitate the therapeutic process. This is a promising solution to the problem of high attrition observed in today's more ‘instructional’ programmes, despite their excellent therapeutic value for those individuals motivated enough to work through the programme. We now anticipate another generation of autonomous online psychotherapy where programmes will become responsive to the circumstances of the individual user and offer an agile, adaptive environment in which the user should feel more personally engaged with the process.

It might be intuitively strange to think about psychotherapy, a highly relational form of medical treatment, being in the forefront of autonomous digital care. Perhaps this can be explained by the fact that we are often seeking to treat the unfortunate effects of earlier interactions with others. Themed interaction with another's mind, through an internet programme, offers a fresh framework through which earlier negative experiences might be reconsidered, reappraised and restructured for future well-being. Ultimately, the future of autonomous digital psychotherapy is not about communication with a computer, but with the body of knowledge established through evidence-based practice and its dynamic tailoring to personal need.

## Supplementary material

For supplementary material accompanying this paper visit https://doi.org/10.1192/bjb.2019.66. click here to view supplementary material

## About the authors

**Annabel McDonald**, MBBS, MRCPsych, is a psychiatry consultant and PhD student at Brighton and Sussex Medical School, UK. **Jessica A. Eccles**, MBBS, PhD, MRCPsych, is a National Institute for Health Research academic clinical lecturer at Brighton and Sussex Medical School, UK. **Sanaz Fallahkhair**, PhD, is a senior lecturer at the School of Computing Engineering and Maths, University of Brighton, UK. **Hugo D. Critchley** is Chair of Psychiatry at Brighton and Sussex Medical School, UK.
