# Supplementary material for: Online psychotherapy: trailblazing digital healthcare
Source: BJPsych Bull. 2019 Oct 7;44(2):60–6. doi: 10.1192/bjb.2019.66 (PMC7283124; doi:10.1192/bjb.2019.66)
Supplement: Supplementary file 1 [file S2056469419000664sup001.docx]

Supplementary Table 1 Effect Sizes and Compliance

| Authors | Therapy | Subjects | Study | *n*= | Scale | Effect Size | Compliance | Additional comments |
| --- | --- | --- | --- | --- | --- | --- | --- | --- |
| Christensen *et al* 2006^1^ | CBT MoodGYM | Web clicks | Trial | 2794 | GDS | 0.4 for those who completed>half of modules | 20.4% completed assigned intervention | Allocated to varying numbers of modules |
| Mackinnon *et al*, 2008 ^2^ | CBT MoodGYM | Community patients with depression | RCT, | 525 | CES-D | 0.38 relative to control | 66% completed treatment | Weekly phone calls from lay interviewers to report on progress and encourage adherence |
| Warmerdam *et al*, 2010^3^ | PST Self-Examination Therapy | adverts &, CES-D > 15 | RCT | 263 | CES-D | 0.47 relative to control | 55.7% completed 3 sessions 37.5% completed all | Email encouragement |
| Warmerdam *et al*, 2010 ^3^ | CBT Coping with Depression | adverts &, CES-D > 15 | RCT | 263 | CES-D | 0.54 relative to control | 71.6% completed half 38.6% completed all | Email encouragement |
| de Graaf *et al*, 2009 ^4^ | CBT Colour Your Life | Random invitations. Screened to have mild-moderate+ depression | RCT | 303 | BDI | Effect size = 0.84 No significant difference to TAU, | 42% completed>5 sessions (seen as ‘adequate dose’) |  |
| Cuijpers *et al*, 2011 ^5^ | CBT |  | Meta-analysis | 7 studies, 1362 users | Various | d = 0.28 compared to control | ‘Use of treatments was low’ eg ‘in one of the studies only 14% completed all sessions’ |  |
| Farrer et al, 2011 ^6^ | CBT MoodGYM | Helpline callers with score >21 on KPDS | Trial | 155 | CES-D | g=0.76 compared to placebo | 31.6% completed > half of modules 15.8% completed all |  |
| Moritz et al, 2012 ^7^ | CBT | adverts | RCT, | 210 | BDI | d=0.36 | Average completion was 53% of sessions | Email reminders used |
| Ruwaard et al, 2012 ^8^ | CBT | GP referrals for psychotherapy | Trial | 1500 | Various | d=1.3 | 71% completed all treatment | Included text messaging with therapist |
| Espie et al, 2012 ^9^ | CBT ‘Prof’ | Great British Sleep Survey insomniacs | RCT | 164 | % time asleep | d=1.06 compared to placebo | 88% completed >4 sessions, 82% completed all | Included support forum & automated emails |
| Griffiths et al, 2012 ^10^ | CBT e-couch | Random invitations. Screened that KPDS>20 | RCT | 311 | caseness | Significantly greater reduction in caseness relative to control | 65% completed all 12 modules, |  |
| Calear et al, 2013 ^11^ | CBT MoodGYM | School adolescents | Trial | 1477 | RCMAS | Relative to W/L: 1. d=0.22 if 0-19 exercises complete 2. d=0.39 if 20-29 exercises done | 36% completed half of the exercises |  |
| Proudfoot et al, 2012 ^12^ | Bipolar Education Program | Adverts for Bipolar users | RCT | 419 | GADS | No significant change relative to controls | 74% completed half 39% completed all |  |
| Christensen et al, 2014 ^13^ | CBT iChill | Random invitations. Screened that GAD7>5 | RCT | 558 | GAD7 | Not significant compared to placebo | 35% attrition | Combinations of CBT, physical activation, relaxation, phone calls, emails, placebo |
| Twomey et al, 2014 ^14^ | CBT MoodGYM | Referrals to psychology | RCT | 149 | DASS-21 | d=0.48 d=0.14 relative to W/L | 27% completed all | Weekly automated emails |
| Menga et al, 2014 ^15^ | CBT MoodGYM | Rheumatology clinic | RCT | 56 | FIQ composite | Intervention score significantly better than TAU |  | Control was TAU |
| Howie & Malouff, 2014 ^16^ | CBT Cognitive Restructuring | Advertising & Trait Anger Scale>21 | RCT | 75 | Trait Anger Scale | d=0.56 relative to waiting list | 65% completed half of modules 30% completed all |  |
| Proyer et al, 2014 ^17^ | Positive psychology | Advertisement | Trial | 510 | AHI | Not significant for individual groups | 378-480/510 | Five groups with different versions of Positive Psychology |
| Murray *et al*, 2015 ^18^ | Mindfulness | Late Bipolar Affect Disorder | Pilot Trial | 26 | QoL.BD | d=0.52 on ITT d=0.72 if competed | 62% completed | No control |
| Karyotaki *et al*, 2015 ^19^ | 8/10 studies were CBT |  | Meta-analysis | 2705 (10 studies) | various |  | 41% completed half 17% completed all |  |
| Zhou *et al*, 2016 ^20^ | CBT | Subthreshold depression | Meta-analysis | 8 RCT’s | various | significantly better than control. SMD = −0.46 | Average drop out rate of 34.5% |  |
| Karyotaki *et al*, 2017 ^21^ | CBT | Depressed subjects | Meta-analysis | 13 studies 3876 | various | g=0.27 relative to control |  | Adherence predicted better outcomes |
| Carlbring *et al*, 2018 ^22^ | CBT | Psychiatric & somatic disorders | Meta-analysis | 20 studies, 1418 | various | g-0.05 relative to therapist CBT |  | ‘equivalent’ effects to face-to-face CBT |
| Ahern *et al*, 2018 ^23^ | CBT | Depression diagnosed | Meta-analysis | 29 studies, 3021 | various | g=0.44 g=0.06 compared to therapist CBT |  | ‘as efficacious as face-to-face CBT’. 24 studies included email or phone calls |

W/L, Waiting List; TAU, Treatment as Usual; CES-D, Centre for Epidemiologic Studies Depression; FIQ, Fibromyalgia Impact Questionnaire; PST, Problem Solving Therapy; ITT, Intention to Treat; QoL.BD, Quality of Life in Bipolar Disorder scale; AHI, Authentic Happiness Inventory; RT, Randomised Trial; G, Hedge’s g; DASS-21, Depression, Anxiety, and Stress Scale-21; CBT, Cognitive Behavioural Therapy; KPDS, Kessler Psychological Distress Scale; BDI, Beck Depression Inventory; GAD7, Generalised Anxiety Disorder 7-item scale; RCT, Randomised Controlled Trial; RCMAS, Revised Children’s Manifest Anxiety Scale; GDS, Goldberg Depression Scale; SMD, Standardised Mean Difference; BDI.PC, Becks depression inventory for primary care; D, Cohen’s d; GADS, Goldberg Anxiety and Depression Scale.

1. Christensen, H.; Leach, L. S.; Barney, L.; Mackinnon, A. J.; Griffiths, K. M., The effect of web based depression interventions on self reported help seeking: randomised controlled trial [ISRCTN77824516]. *BMC psychiatry* **2006,** *6*, 13.

2. Mackinnon, A.; Griffiths, K. M.; Christensen, H., Comparative randomised trial of online cognitive-behavioural therapy and an information website for depression: 12-month outcomes. *The British Journal of Psychiatry* **2008,** *192* (2), 130-134.

3. Warmerdam, L.; van Straten, A.; Jongsma, J.; Twisk, J.; Cuijpers, P., Online cognitive behavioral therapy and problem-solving therapy for depressive symptoms: Exploring mechanisms of change. *Journal of behavior therapy and experimental psychiatry* **2010,** *41* (1), 64-70.

4. de Graaf, L. E.; Gerhards, S. A. H.; Arntz, A.; Riper, H.; Metsemakers, J. F. M.; Evers, S. M. A. A.; Severens, J. L.; Widdershoven, G.; Huibers, M. J. H., Clinical effectiveness of online computerised cognitive-behavioural therapy without support for depression in primary care: randomised trial. *The British journal of psychiatry : the journal of mental science* **2009,** *195* (1), 73-80.

5. Cuijpers, P.; Geraedts, A. S.; van Oppen, P.; Andersson, G.; Markowitz, J. C.; van Straten, A., Interpersonal psychotherapy for depression: a meta-analysis. *American Journal of Psychiatry* **2011,** *168* (6), 581-592.

6. Farrer, L.; Christensen, H.; Griffiths, K. M.; Mackinnon, A., Internet-based CBT for depression with and without telephone tracking in a national helpline: randomised controlled trial. *PloS one* **2011,** *6* (11), 1.

7. Moritz, S.; Schilling, L.; Hauschildt, M.; Schröder, J.; Treszl, A., A randomized controlled trial of internet-based therapy in depression. *Behaviour Research and Therapy* **2012,** *50* (7), 513-521.

8. Ruwaard, J.; Lange, A.; Schrieken, B.; Dolan, C. V.; Emmelkamp, P., The effectiveness of online cognitive behavioral treatment in routine clinical practice. *PLoS One* **2012,** *7* (7), e40089.

9. Espie, C. A.; Kyle, S. D.; Williams, C.; Ong, J. C.; Douglas, N. J.; Hames, P.; Brown, J. S. L., A randomized, placebo-controlled trial of online cognitive behavioral therapy for chronic insomnia disorder delivered via an automated media-rich web application. *Sleep: Journal of Sleep and Sleep Disorders Research* **2012,** *35* (6), 769-781.

10. Griffiths, K. M.; Mackinnon, A. J.; Crisp, D. A.; Christensen, H.; Bennett, K.; Farrer, L., The effectiveness of an online support group for members of the community with depression: a randomised controlled trial. *PloS one* **2012,** *7* (12), 1.

11. Calear, A. L.; Christensen, H.; Mackinnon, A.; Griffiths, K. M., Adherence to the MoodGYM program: outcomes and predictors for an adolescent school-based population. *Journal of affective disorders* **2013,** *147* (1-3), 338-344.

12. Proudfoot, J.; Parker, G.; Manicavasagar, V.; Hadzi-Pavlovic, D.; Whitton, A.; Nicholas, J.; Smith, M.; Burckhardt, R., Effects of adjunctive peer support on perceptions of illness control and understanding in an online psychoeducation program for bipolar disorder: A randomised controlled trial. *Journal of Affective Disorders* **2012,** *142* (1), 98-105.

13. Christensen, H.; Batterham, P.; Calear, A., Online interventions for anxiety disorders. *Curr Opin Psychiatry* **2014,** *27* (1), 7-13.

14. Twomey, C.; O'Reilly, G.; Byrne, M.; Bury, M.; White, A.; Kissane, S.; McMahon, A.; Clancy, N., A randomized controlled trial of the computerized CBT programme, MoodGYM, for public mental health service users waiting for interventions. *The British journal of clinical psychology* **2014,** *53* (4), 433-450.

15. Menga, G.; Ing, S.; Khan, O.; Dupre, B.; Dornelles, A. C.; Alarakhia, A.; Davis, W.; Zakem, J.; Webb-Detiege, T.; Scopelitis, E.; Quinet, R., Fibromyalgia: can online cognitive behavioral therapy help? *The Ochsner journal* **2014,** *14* (3), 343-349.

16. Howie, A. J.; Malouff, J. M., Effects of online cognitive treatment for problematic anger: a randomized controlled trial. *Cogn Behav Ther* **2014,** *43* (4), 310-8.

17. Proyer, R. T.; Gander, F.; Wellenzohn, S.; Ruch, W., Positive psychology interventions in people aged 50-79 years: long-term effects of placebo-controlled online interventions on well-being and depression. *Aging Ment Health* **2014,** *18* (8), 997-1005.

18. Murray, G.; Leitan, N. D.; Berk, M.; Thomas, N.; Michalak, E.; Berk, L.; Johnson, S. L.; Jones, S.; Perich, T.; Allen, N. B.; Kyrios, M., Online mindfulness-based intervention for late-stage bipolar disorder: pilot evidence for feasibility and effectiveness. *J Affect Disord* **2015,** *178*, 46-51.

19. Karyotaki, E.; Kleiboer, A.; Smit, F.; Turner, D. T.; Pastor, A. M.; Andersson, G.; Berger, T.; Botella, C.; Breton, J. M.; Carlbring, P.; Christensen, H.; de Graaf, E.; Griffiths, K.; Donker, T.; Farrer, L.; Huibers, M. J. H.; Lenndin, J.; Mackinnon, A.; Meyer, B.; Moritz, S.; Riper, H.; Spek, V.; Vernmark, K.; Cuijpers, P., Predictors of treatment dropout in self-guided web-based interventions for depression: an ‘individual patient data’ meta-analysis. *Psychological medicine* **2015,** *45* (13), 2717-2726.

20. Zhou, T.; Li, X.; Pei, Y.; Gao, J.; Kong, J., Internet-based cognitive behavioural therapy for subthreshold depression: a systematic review and meta-analysis. *BMC psychiatry* **2016,** *16* (1), 356.

21. Karyotaki, E.; Riper, H.; Twisk, J.; Hoogendoorn, A.; Kleiboer, A.; Mira, A.; Mackinnon, A.; Meyer, B.; Botella, C.; Littlewood, E.; Andersson, G.; Christensen, H.; Klein, J. P.; Schröder, J.; Bretón-López, J.; Scheider, J.; Griffiths, K.; Farrer, L.; Huibers, M. J. H.; Phillips, R.; Gilbody, S.; Moritz, S.; Berger, T.; Pop, V.; Spek, V.; Cuijpers, P., Efficacy of Self-guided Internet-Based Cognitive Behavioral Therapy in the Treatment of Depressive Symptoms: A Meta-analysis of Individual Participant Data. *JAMA psychiatry* **2017,** *74* (4), 351-359.

22. Carlbring, P.; Andersson, G.; Cuijpers, P.; Riper, H.; Hedman-Lagerlöf, E., Internet-based vs. face-to-face cognitive behavior therapy for psychiatric and somatic disorders: an updated systematic review and meta-analysis. *Cognitive Behaviour Therapy* **2018,** *47* (1), 1-18.

23. Ahern, E.; Kinsella, S.; Semkovska, M., Clinical efficacy and economic evaluation of online cognitive behavioral therapy for major depressive disorder: a systematic review and meta-analysis. *Expert review of pharmacoeconomics & outcomes research* **2018,** *18* (1), 25-41.
